# Using Virtual Reality Interventions to Promote Social and Emotional Learning for Children and Adolescents: A Systematic Review and Meta-Analysis

**DOI:** 10.3390/children11010041

**Published:** 2023-12-29

**Authors:** Feng Zhang, Yan Zhang, Gege Li, Heng Luo

**Affiliations:** Faculty of Artificial Intelligence in Education, Central China Normal University, Wuhan 430079, China; zhangfeng77@mails.ccnu.edu.cn (F.Z.); zhangyan123@mails.ccnu.edu.cn (Y.Z.); ligg323@mails.ccnu.edu.cn (G.L.)

**Keywords:** social and emotional learning, virtual reality, children, systematic literature review, meta-analysis

## Abstract

This study provides a comprehensive review of the application of virtual reality (VR) in social and emotional learning (SEL) for children and adolescents over the past decade (January 2013–May 2023), with a specific interest in the relations between their technological and instructional design features. A search in Web of Science resulted in 32 relevant articles that were then manually screened. Coding analysis was conducted from four perspectives: participant characteristics, research design, technological features, and instructional design. The analysis provides insights into the VR literature regarding publication trends, target populations, technological features, instructional scenarios, and tasks. To test the effectiveness of VR interventions for promoting SEL, a meta-analysis was also conducted, which revealed an overall medium effect size and significant moderating effects of SEL disorder type and instructional task. Finally, based on the research results, the practical implications of and future research directions for applying VR in SEL were discussed.

## 1. Introduction

In recent years, social and emotional learning (SEL) has received attention around the world. SEL is defined as “the process through which all young people and adults acquire and apply the knowledge, skills, and attitudes to develop healthy identities, manage emotions and achieve personal and collective goals, feel and show empathy for others, establish and maintain supportive relationships, and make responsible and caring decisions” [1,2,3]. SEL promotes attention to students’ emotional and social needs, respect for their individuality and differences, and the development of their self-awareness and social skills, which is consistent with competency-oriented pedagogy and education in the concept of post-metaphysical thinking [4,5]. Research has found that social and emotional competence can predict a child’s mental health [6], while a lack of such competence is strongly associated with behaviors such as suicide, as poor behavioral and emotional control is the leading cause of death among adolescents [7]. SEL has also been related to children’s academic performance [8,9] because children with strong social and emotional skills can set their own goals, manage stress, find good ways to study, and use interpersonal skills to solve problems [10]. Over time, children with SEL abilities can act according to their own values, care about others, and take responsibility for their own behaviors [11]. Families and schools have a major impact on the development of values in children and adolescents, while the media also exert a significant influence [12].

With the rapid development of high-speed communication and mobile technology, virtual reality (VR) has become an effective way to improve SEL. VR is the next generation of interactive display technology that provides a sense of presence [13]. A large number of VR devices have appeared at affordable prices in recent years, and the software and hardware problems are being solved [14,15]. Whether it is app-based desktop VR or immersive VR based on a head-mounted display (HMD), VR allows the user interaction with the environment through the input device [16]. VR can connect the physical world with a virtual environment, pursue high-fidelity simulation of real life, and eliminate the sense of mediation [17].

Studies have shown that interventions using VR can improve social skills and emotional recognition in children and adolescents [18,19]. Compared with traditional SEL interventions, VR interventions have several unique advantages. First, VR intervention groups are more targeted. There are VR interventions not only for typically developed (TD) children [20] but also for children with neurological disorders [21]. School-based intervention projects have focused on the role of the environment and the use of generic SEL curricula [22,23]. However, this type of school-based SEL approach addresses the entire student body rather than intervening with a specific group [24]. Children with neurodevelopmental disorders and behavioral problems have greater challenges with SEL [25]. Second, VR can provide realistic life scenarios that allow people interaction with technology, thus providing an immersive feeling. Third, VR provides a safe and controllable social response environment, helps children learn new knowledge and skills, and can be applied to scenes similar to those that occur in daily life [26,27].

VR has rich potential to promote SEL, and studies have reviewed the use of VR for teaching social skills [28], emotion recognition [29], and life skills [30]. However, these reviews focused on special populations such as those with autism spectrum disorder (ASD), intellectual disabilities, and social anxiety disorder (SAD), and did not find a single review for TD children. Studies have focused on exceptional children, which makes it unclear how effective VR is in assisting TD children. Many reviews have also limited participants by a particular disorder type and did not focus on age [16,31], which means that experimental results cannot be analyzed based on age-specific developmental characteristics. A comprehensive review of all types of children and adolescents is therefore necessary to clarify the role of VR for SEL.

While several studies have systematically reviewed the use of VR in SEL in children and adolescents, they have not focused on the features of VR itself. For example, Mesa-Gresa et al. [32] only recorded the VR hardware and software devices used, and the research results were described based on different dimensions of SEL, with less analysis of VR. Adabla et al. [33] described the types of VR equipment, scenarios, and tasks in the reviewed articles but did not analyze the interaction of VR or provide statistical data, so it was impossible to clearly understand the ratio for the application of VR with different features. The lack of a comprehensive analysis of VR in review articles makes it difficult to explore what kind of VR is most widely used, as well as what characteristics of VR are most effective in promoting SEL in children and adolescents.

To address this research gap, this study reviewed the empirical research literature on the application of VR to SEL in children and adolescents over the past 10 years (2013–2023) and systematically analyzed the applied research trends in terms of research design, technical features, instructional design, and teaching effects. This review sought to describe the features of VR more comprehensively, summarize the specific types of SEL promoted by VR, and answer the following research questions:

1. Which groups were primarily targeted when using VR to promote SEL?
2. What are the technical features of VR that support SEL for children and adolescents?
3. What types of social and emotional skills for children and adolescents were taught in VR?
4. What are the overall effects and possible moderating factors of VR-supported SEL interventions?

## 2. Methods

Articles and related information were obtained according to the PRISMA protocol, which included assessing whether the articles met the requirements for analysis according to specific standards. This process is divided into three parts: search, screening, and coding. This study followed the Preferred Reporting Items for Systematic Reviews and Meta-Analysis statement for the selection and use of research methods. The protocol for this study was registered with INPLASY (2023110115).

### 2.1. Search Procedure

A literature search was conducted according to the standard systematic literature review process. Articles were retrieved from the core collection of the Web of Science online database to ensure the quality of the article. Articles were limited to those written in English and published between January 2013 and May 2023. We chose the year 2013 as the start of our literature search since it witnessed the release of Oculus Rift DK1, which marked the successful commercialization of VR devices featured by advanced functions and low cost. The devices provide people with more opportunities to experience VR. They also enable developers to create VR applications with greater ease and more diversity. As a result, we decided to start literature search in 2013. The following search terms were used: (a) “virtual reality” or “VR” or “virtual” or “Immersive Virtual Environment” or “Immersive Technolog*” or “3D Environment” or “cave” (because cave environments are not clearly classified by level of immersion, some articles use the term “cave” in their titles instead of “VR”); (b) “social emotional learning” or “social emotional competence” or “social emotional development” or “social emotional skill*” or “social skill*”; and (c) “child*” or “youth” or “young people” or “teenager” or “adolescent”. Using these search criteria, a total of 503 articles were retrieved.

### 2.2. Screening Procedure

In this phase, the titles and abstracts of the articles were read, and two duplicate articles were deleted. The three authors then discussed the inclusion and exclusion criteria in detail, as shown in Table 1. We first screened the titles and abstracts of retrieved articles and manually excluded review and non-empirical research articles. If the title and abstract clearly indicated that no SEL intervention for children and adolescents with VR was used, the articles were excluded. We retained articles when the title and abstract information was insufficient to determine whether the exclusion criteria were met; a total of 441 articles were excluded in this step.

**Figure 1:**
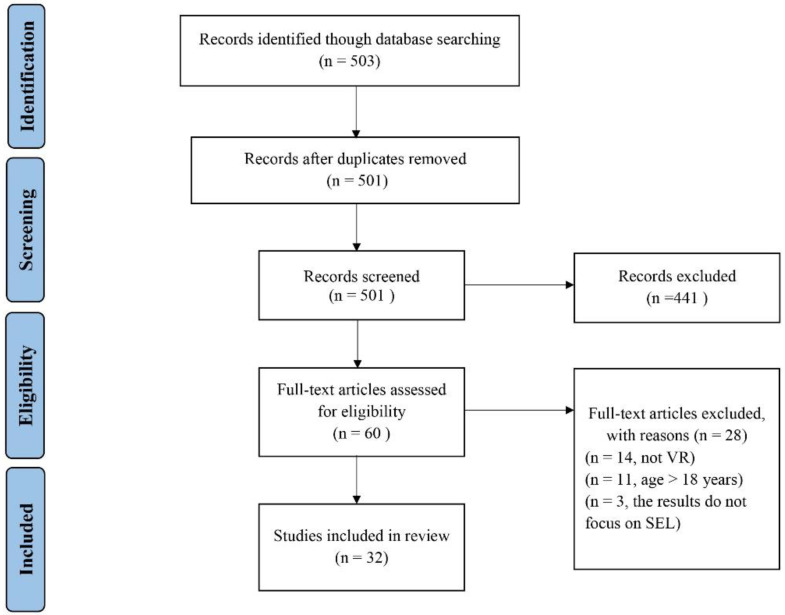
Flow diagram of the study selection process.

The full texts of the remaining 60 articles were read in a secondary screening to ensure that the articles met all of the inclusion criteria. The first criterion was that the participants were younger than 18 years of age, followed by the requirement that the participants engaged in SEL in a VR environment, and that attention was paid to the impact of VR on improving SEL. As shown in Figure 1, after all screening was complete, a total of 32 articles met the criteria and were included in the analysis.

### 2.3. Coding Procedure

As shown in Table 2, we recorded five categories of specific information for the articles that were ultimately included: article metadata, participant characteristics, research design, technological features, and instructional design. The metadata include basic information such as the authors, year of publication, region, and more. For the participant characteristics, in addition to age, gender, and grade level, intelligence quotient (IQ) and disorder type were also recorded, as most of the participants had ASD, attention deficit, and other disorders. The research type, sample size, number of interventions, and study duration were recorded in the study design. In the technological features, the characteristics of hardware equipment and technology were recorded, and VR was divided into three types: virtual world, simulation, and game [34]. Finally, instructional design was recorded.

After deciding on the coding dimension, the first and second authors coded five articles and discussed any uncertainties during the coding process in detail to ensure a common understanding between the two coders. The first and second authors then coded separately, with the first round of coding being completed within 2 weeks. During the coding process, the two coders remained in constant communication and jointly resolved any coding uncertainties when they arose. During the coding process, any areas that were difficult for both coders to determine were recorded and then subsequently discussed with an expert. Following expert guidance, both coders undertook secondary coding. This primarily involved addressing any gaps in the coding table and ensuring the accuracy of the previous coding. The entire coding process lasted one month. Cohen’s Kappa was calculated to measure the inter-rater agreement among coders, and the Kappa value of 0.817 suggests an overall good inter-rater reliability for the coding results.

Moreover, we conducted a meta-analysis of experimental research published between 2013 and 2023 to understand the overall effectiveness of VR for SEL in children and adolescents. Multiple articles included more than one dependent variable related to SEL, and we treated these as separate studies. A total of 31 studies from 15 articles reported the statistics needed to calculate the effect size. Sample size (N), mean value (Mean), standard deviation (SD), *p*-value, or *t*-value were extracted from the experimental group and the control group or before and after for a single group in the study. We used comprehensive meta-analysis (CMA version 3.0) software for analysis, and Hedge’s *g* was chosen for the final effect value [35].

## 3. Results

**Figure 2:**
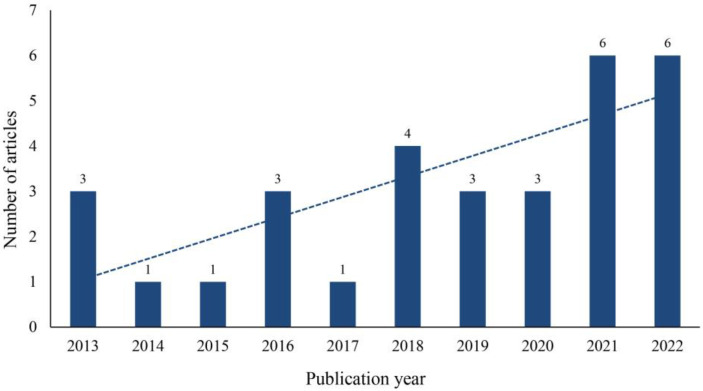
Number of articles published.

Based on the collected article metadata, 32 articles were analyzed in total. As shown in Figure 2, from 2013 to 2022 (note that 2023 is not included because data for the full year were not available), the number of published articles followed an overall upward trend. One possible reason that the number of studies peaked in 2021 is that the COVID-19 pandemic caused more students to have social and emotional problems [36]. Of the 32 articles, 5 were conference papers, 3 of which were from *IEEE Transactions on Neural and Rehabilitation Engineering,* accounting for 60% of conference papers. There were 27 journal papers, of which the highest ranked source was *Computers and Education*, with 3 papers. Among the 25 studies that reported the location of the experiments, the United States ranked first, accounting for 48% (n = 12) and China ranked second (n = 5, including one each in Hong Kong and Taiwan).

### 3.1. Participant Characteristics

According to screening criteria, participants were between the ages of 4 and 18. There was one study in which only one of the nine participants was over the age of 18; this article was included at the authors’ discretion. Out of all of the studies, 44% included participant populations that were mostly male (male participants made up more than two-thirds of the total, n = 14), 34% had a gender balance (n = 11), and 9% were all male (n = 3). This is similar to the findings of Li et al. [37] and Mesa-Gresa and Gil-Gómez [32], where the study populations tended to include more male participants. Furthermore, only half of the studies reported grade levels, two were of preschool age, nine were for primary school children, two were for secondary school students, and three were for both primary and secondary school students. There were no studies specifically on secondary school students, perhaps because psychology experiments tend to focus on age rather than grade level.

The participants’ disorder types were divided into five categories: ASD, TD, mixed (two disorders mixed), social anxiety disorder, and other. Other included participants with neurological disorders, and other social problems. There were 20 studies in which participants had ASD (63%). Only four of the studies had participants who were free of any disorders. Because most of the participants had disorders, we recorded their IQ values. A total of 16 studies reported the IQ of the participants, 14 of which had an IQ of 80 or more, one of which had an IQ of 80 or less, and one of which included both those with an IQ of 80 or more and those with an IQ of 80 or less. In the literature search, 88% of the study participants were found to have disorders. This may be because children with disorders have more severe deficits in social and emotional competence, which has thus attracted the attention of researchers.

### 3.2. Research Design

Among the 32 articles, there were 29 experimental studies, one mixed study, one survey study, and one case study. All experimental studies used a pre–post design, and seven had an intervention group and a control group. Research has mostly focused on changes in children who use VR to learn social and emotional skills rather than comparisons between the different effects of VR and other technologies in promoting SEL. The mixed study collected quantitative data and interviewed teachers to assess how students apply what they learn in VR to real-life situations [38]. The survey study was conducted to investigate the feasibility of a virtual environment program through the experiences of 11 children with social anxiety disorder in a virtual school. The study data and user comments were then fed back to the development team to refine subsequent software revisions [39]. In the case study, a child with ASD and a child with attention deficit hyperactivity disorder had social interactions in a safe virtual fish shop, and the researchers evaluated the effectiveness of VR through the children’s conversations and interactions with their mothers [40]. In addition, consistent with the findings reported by Irish [41], this review also observed a lack of longitudinal studies, with only four studies featuring follow-up.

**Figure 3:**
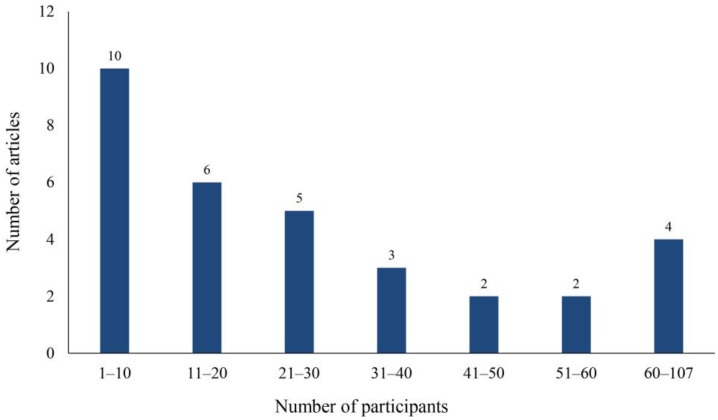
Sample size distribution map.

The largest number of participants was 107 in a study that recruited children with ASD in Hong Kong to develop their social and emotional competence through practices in a simulated school scenario. The results showed that children in the VR intervention group had significantly higher abilities in all aspects than those in the control group [42]. The smallest sample size was only two people, which was also the only case study. This study recorded the field performance and interview data of two participants, as well as analyzed and discussed the feasibility of the VR system [40]. As shown in Figure 3, there were 24 studies with a sample size of less than 40 people, accounting for 75% of the studies reviewed. In summary, the sample sizes for the reviewed studies were relatively small, which is consistent with the findings of Lorenzo et al. [43]; Newbutt et al. [44] suggested, however, that research findings based on small sample sizes can be limited.

**Figure 4:**
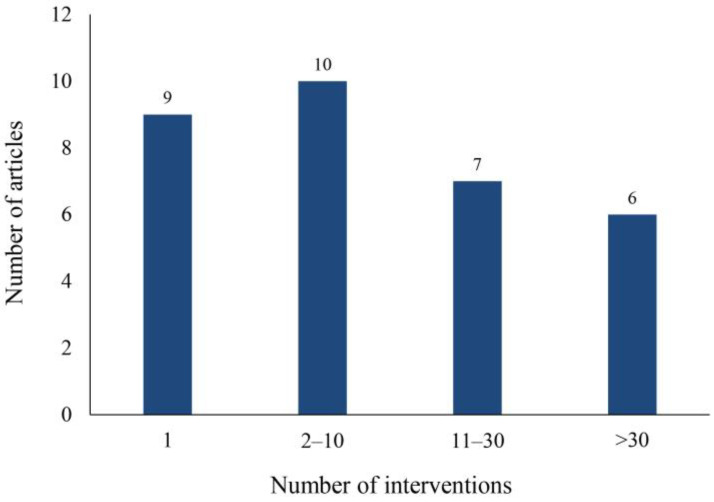
Intervention frequency chart.

As shown in Figure 4, nine studies administered only one intervention. Ten studies contained 2–10 interventions, and there were six studies with more than 30 interventions. The shortest intervention duration was 15 min and the longest was more than 60 h. During the 15 min intervention, children exercised their social skills using an HMD to enter a virtual store, identify items on the shelves, and look at the owner who helped them make purchases. The study did not quantify children’s learning outcomes but focused on children’s experiences in VR [40]. The longest intervention lasted for three months and consisted of 90 45-min sessions. This study divided participants into three groups, VR, VR and medication, and control, and was the only study that used medication [45]. Most of the studies (72%) involved two or more interventions, possibly because a single intervention is rarely effective for a child with a disorder.

In combination with the number of interventions and the duration of intervention, studies on the use of HMD and VR glasses in long-term interventions have ranged from 25 to 45 min per intervention, and the use of such devices in children can have effects such as dizziness, which thus limits the duration of such interventions. Two other studies of long-term interventions used projectors to watch videos and VR software downloads to study at home [39,46]. Instead of conducting the intervention near the researcher, it was more convenient for the participants to go home and use the software to learn; however, there are many influencing factors, and the downloaded resources could only carry out simple and repeated interactions.

**Figure 5:**
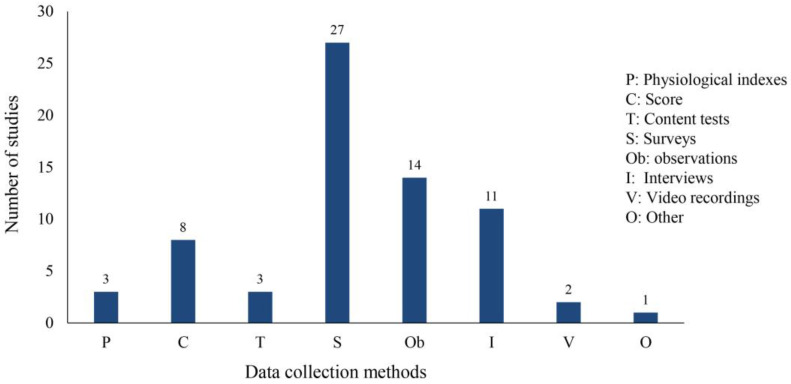
Data collection methods.

The included studies used seven ways to collect data: physiological indexes, such as eye movement, pulse volume map, and skin temperature signals; scores in VR games or scene missions; content tests, including card, emotion recognition, and question tests; surveys, in which data were collected using questionnaires; observations, when participants’ reactions were recorded by the researcher or their parents’ observations; interviews of students, parents, or teachers; video recordings of the intervention process; and others, such as the use of the Avatar protocol to collect student development information. Each study used one or more of these methods to collect data. As shown in Figure 5, the most commonly used data collection method was the questionnaire survey (n = 27). It is worth noting that three studies collected physiological indicators [20,47,48], and collecting considerable physiological data in combination with other subjective data such as questionnaires or observations can better reflect the real state of the participants.

### 3.3. Technological Features

**Figure 6:**
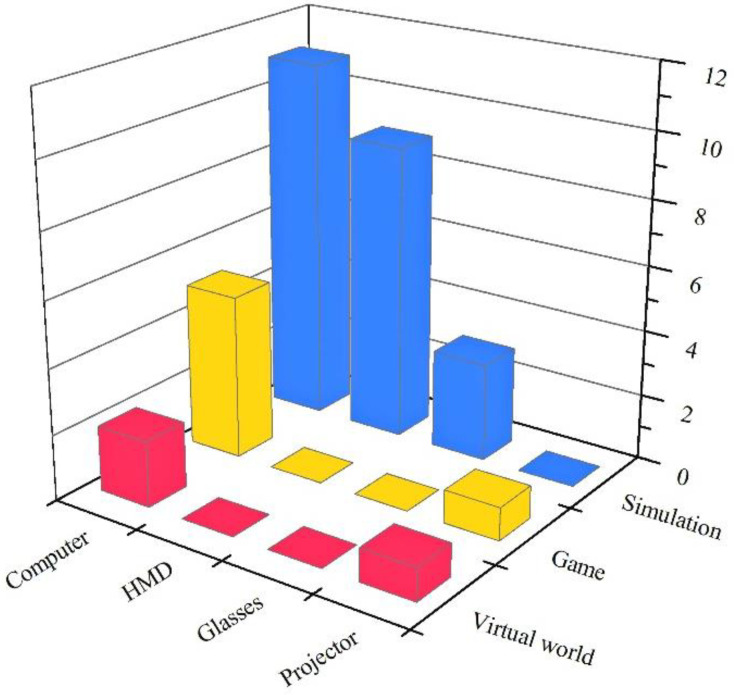
Number of studies by type of equipment and technology.

According to the definition by Merchant and Goetz [34], VR technology can be divided into three categories: simulation, games, and virtual worlds. As shown in Figure 6, simulation ranked first, followed by games and virtual worlds. Unfortunately, only three virtual worlds were studied in the application of VR to SEL. Most VR was preset in advance, and there was no real open communication. Four types of VR equipment were used: (1) computers, used in 18 studies; (2) HMD, used in nine studies; (3) glasses, used in three studies; and (4) projectors, used in two studies (the equipment used by the cave automatic virtual environment was a projector). Among the 12 studies that employed HMD and glasses, full immersion was observed, while the remaining 20 studies were semi-immersive. For example, in the study conducted by He et al. [49], children were able to experience an updated virtual environment through HMD, and engaged in various pedagogical activities such as social interactions, object placement, and basketball shooting within this immersive environment. Notably, this learning process was intentionally detached from the real world to achieve complete immersion [50]. In contrast, the use of semi-immersive technology enables children to engage with the physical world while carrying out instructional tasks within a simulated setting. For example, in the study by Tsai et al. [51], therapists provided guidance to children as they engaged in emotional recognition in a cave environment.

As shown in Figure 6, the most extensively researched method involved the simulation of scenarios through computing equipment. The second method employed HMD for interactive engagement with simulated environments. The third method employed computers to develop social and emotional competence in the form of games. From an equipment standpoint, the contrast between children using computers and HMD for learning experiences in the simulated environment was primarily based on immersion and operational methods, as previously noted. Considering the technology type, children have different goals and tasks when using computer equipment to participate in virtual scenes and play games. The simulations aimed to recreate the real world and allow students practice of social skills, such as greeting people and expressing their opinions [52]. By contrast, games were more entertaining and helped students cooperate with others and regulate their emotions in the context of storylines and motivational points [53,54]. The use of virtual worlds and game-type VR also tended to exclude HMD and glasses equipment, as shown in Figure 6, which indicates the current limitations of semi-immersive environments. Virtual worlds enhance interaction while games garner greater interest from children, potentially yielding improved outcomes when utilized with fully immersive VR technologies.

**Figure 7:**
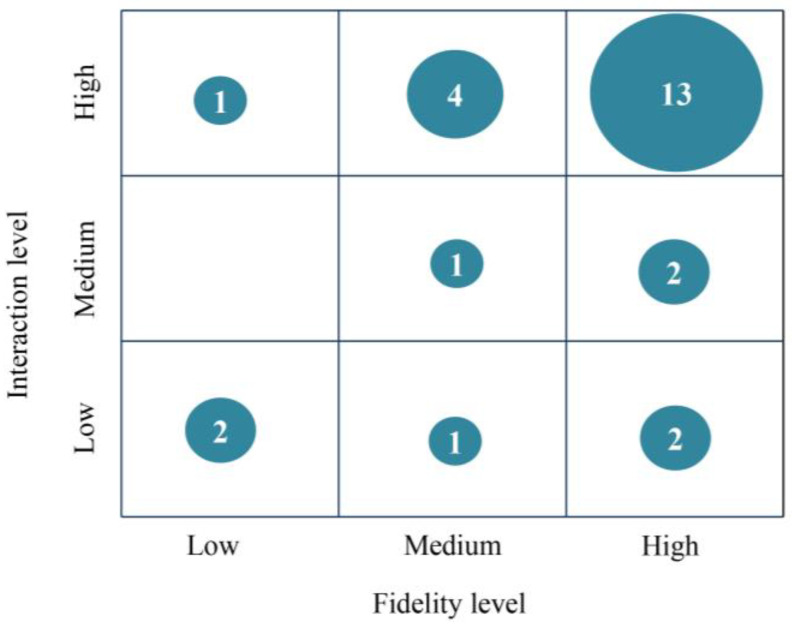
Fidelity and interaction levels.

The fidelity level was judged according to the degree to which reality was simulated. Based on the pictures provided in the text and the author’s description, those that were highly similar to real life were considered as high level, while those with cartoon or mismatched characters were low level. We also considered high-level interactions that permit users to navigate the environment with autonomy or experience motion [55,56], whereas interactions consisting of predetermined conversations or mere clicks were low-level [57,58]. As shown in Figure 7, the double high level received the most attention, followed by VR combinations with high levels of interaction and medium levels of fidelity. VR that combines high-level interaction and high-level fidelity offers children a more practical and authentic setting for SEL. For example, Sarver and Beidel [39] employed exquisite modeling techniques to depict the school setting, accurately capturing the facial expressions and actions of teachers, classmates, school bullies, and other characters. The VR system also provided three levels of interaction with varying degrees of difficulty, which enabled children to engage in learning activities such as greetings, responding to others’ questions, and initiating conversations as they worked to enhance their social skills. The high-fidelity representation and infinite conversational possibilities of the VR system enabled children to socialize freely within the virtual environment.

High-fidelity scenes are more effective in immersing individuals into the created situation, while lower interactions afford only specific conversations and simple click-to-watch interactions, which indicates that this VR combination places greater emphasis on the significance of the environment. According to Lahiri and Bekele [48], for instance, when the avatar tells its own story, VR presents a scene that corresponds to the narrative. The child is then required to engage in a restricted text conversation with the avatar based on the topic suggested by the system. The system gauges how long the child gazes at the avatar’s face, and if the duration is too long, a warning is issued to teach the child the correct pattern of visual engagement during social conversations. In contrast, the combination of low-fidelity and high-interaction VR places greater emphasis on the children’s behavior within the system. In the study by Amat and Zhao [20], the scene consisted solely of a human avatar with a neutral expression against a solid color background; children were required to interact with the avatar visually and obtain puzzle pieces based on the direction of the avatar’s gaze. Children engaged in gaze sharing and gaze following activities by completing jigsaw puzzles, thereby enhancing their social skills.

### 3.4. Instructional Design

**Figure 8:**
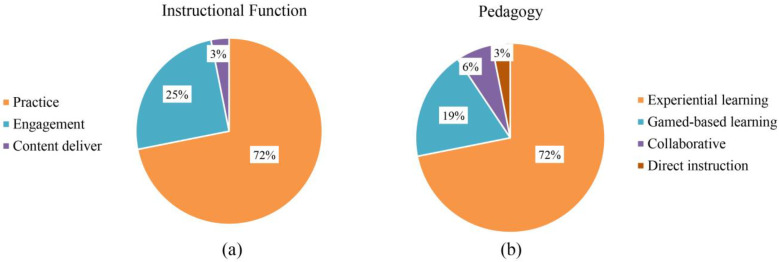
(**a**) Type of instructional function; (**b**) Type of pedagogy.

Instructional functions in the VR interventions included practice, content delivery, and engagement. As shown in Figure 8a, the most common use of VR was to practice social and emotional skills, followed by encouraging students to be more engaged, and the least used was content delivery. Most participants were students with mental disorders, in that they lacked social and emotional competence; thus, it was reasonable to use VR for a large proportion of practice, because simply using VR to present content may not allow students to learn actionable knowledge. As shown in Figure 8b, there were four categories of pedagogies in the studied interventions, with experiential learning being the most commonly adopted, game-based learning the second most popular, and direct instruction being the least utilized. Experiential learning permits students to personally encounter social situations and learn communication and collaboration skills by interacting with the environment and other characters, in addition to applying to the development of children’s social and emotional competence.

**Figure 9:**
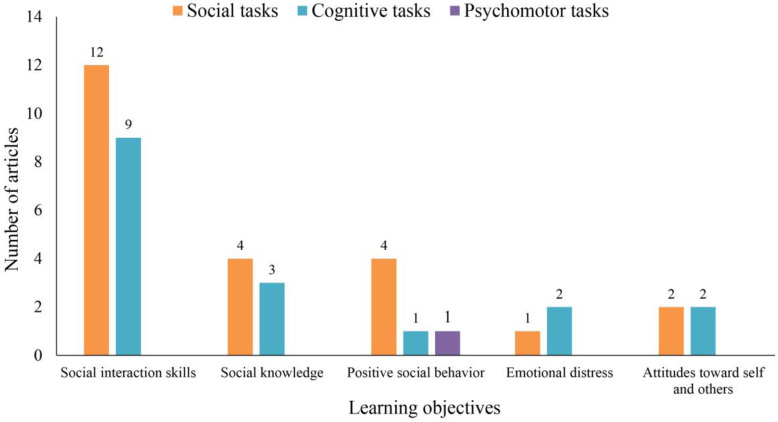
Learning objectives and tasks.

Following Dechsling et al. [59] and Durlak and Weissberg [11], we classified learning objectives into five categories: social interaction skills, including joint attention, communication, and cooperation; social knowledge, consisting of emotion recognition and social understanding; positive social behavior, such as sharing, enjoyment, and anger control; emotional distress, including depression, anxiety, and social phobia; and attitudes toward self and others, including self-esteem, self-efficacy, and sympathy. As shown in Figure 9, social interaction skills accounted for the largest proportion of interventions, which is consistent with the results of Fernández-Sotos et al. [60], followed by social knowledge. The smallest proportion constituted emotional distress and attitudes toward self and others. This result indicated that most research has centered on communication and cooperation in SEL but has paid less attention to self-concept and stress. One possible reason for this is that the explicit behavioral manifestations of children’s lack of social and emotional competence are often related to their difficulties in communicating with others, and the degree of understanding of their own emotions and stressors is more difficult to identify.

Instructional tasks were classified into cognitive tasks (including low-level, high-level, and metacognitive tasks), social tasks, and psychomotor tasks. Low-level cognitive tasks involved memorization, understanding, and simple application of social knowledge, while high-level cognitive tasks required problem-solving abilities. Social tasks involved communication with others, and psychomotor tasks involved following others’ instructions to perform certain actions. It is worth noting that there was only one higher cognitive task and no metacognitive tasks in any of the studied interventions. We believe this is reasonable because the majority of the study participants had mental disorders, which suggests that they would have difficulty performing high-level cognitive tasks. According to Figure 9, the use of social tasks to achieve the instructional objectives of social interaction skills has been extensively studied. This may be because social interaction skills are used extensively in daily life and work, and the performance of social tasks plays a significant role in promoting the psychological development of children and adolescents, as well as enhancing their social adaptability.

The instructional scenarios created by VR technology included physical worlds such as schools, parks, rooms, clinics, natural environments, and buses, as well as games. The instructional scenarios of 24 studies were simulations from real life; only 4 studies focused on games, and 1 contained a mixture of real life and games. Given that children under the age of 18 spend most of their time in school, research has focused on teaching children how to socialize in school situations. Social interaction on the bus was also suggested by children’s parents and teacher, because this environment requires more social interaction [61]. Such social skills need to be learned in real-life situations, which corresponds to the proportion of instructional scenarios. Therefore, when designing VR systems and implementing instruction, the theoretical basis is very important, but in the process of coding, only six articles mentioned frameworks, models, concepts, and theories. This finding aligns with those reported by Li and Belter [37].

### 3.5. Meta-Analysis Results

The results are shown in Table 3. The heterogeneity test proved that there was heterogeneity among the samples (Q = 120.053, *p* < 0.01). According to Higgins et al. [62], *I*^2^ values of 25%, 50%, and 75% represent low, medium, and high heterogeneity, respectively. The Q value and I^2^ indicated that studies have a large degree of heterogeneity (*I*^2^ > 75%), so we calculated the standardized effect sizes using random effects models (REM). According to criteria of Cohen [63], *g* ≤ 0.2, 0.2 < *g* ≤ 0.8, and *g* > 0.8 represent small, medium, and large effects, respectively. As can be seen from Table 3, *g* = 0.378, *p* < 0.001. Therefore, VR has a significant medium positive impact on SEL in children and adolescents.

To determine the factors that affect SEL in children and adolescents, we analyzed the moderating variables, and the results are shown in Table 4. There was no heterogeneity in the number of interventions (*p* > 0.05). Interventions that occurred 2–10 times with more than 30 numbers had a significant promoting effect (*p* < 0.05), and interventions that occurred more than 30 times had the largest effect size (*g* = 0.698). The regulatory effect of only one intervention and 11–30 interventions did not reach a significant level (*p* > 0.05), thus making it unclear whether there was a significant effect on SEL in children and adolescents. There was heterogeneity in disorder types (*p* < 0.001): VR had a negative effect on SAD and a significant positive effect on children with and without other disorders.

From the perspective of VR features, there was no heterogeneity in technology type, hardware equipment, interaction level, and fidelity of simulation. The use of virtual world technology and projection equipment had a large effect size on the promotion of SEL in children and adolescents (*g* = 0.822). Surprisingly, the effect size was the largest for no interaction (*g* = 0.822) and the smallest for high-level interaction (*g* = 0.312). The same is true for fidelity: the effect size of high fidelity (*g* = 0.348) was smaller than that of no fidelity (*g* = 0.483), and the moderating effect of low fidelity was not significant. This result shows that VR of different types, interaction levels, and fidelity can improve SEL among children and adolescents. In other words, more sophisticated techniques do not necessarily lead to better instructional results.

From the perspective of instructional design, there was no heterogeneity in the moderating effect of theoretical foundation, and both theoretical foundation and non-theoretical foundation played a significant promoting role. The effect size with a theoretical foundation (*g* = 0.545) was larger than without (*g* = 0.346). There was also heterogeneity in instructional tasks (*p* = 0.01). High-level cognitive tasks had significant negative effects, while social and mixed tasks had significant positive effects, with social tasks having the largest effect size (*g* = 0.622). The moderating effects of low-level cognitive tasks and psychomotor tasks were not significant. VR for high-level cognitive tasks may have placed an excessive cognitive load on students, which resulted in reduced social and emotional competence. In future VR interventions, more social tasks should be designed, and high-level cognitive tasks should be avoided to promote SEL.

**Figure 10:**
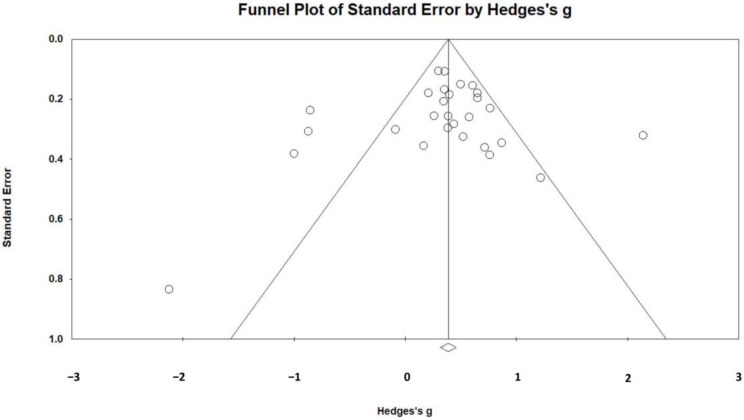
Publication bias funnel plot of effect values.

As shown in Figure 10, most of the study effect values were distributed at the top of the funnel plot, and symmetrical distribution on both sides of the center line proved that the possibility of publication bias was small. At the same time, the classic fail-safe N test was 706, which is higher than the 5*k* (*k* = 31) + 10 proposed by Rosenthal [64]. In summary, there appeared to be no bias in the meta-analysis, and the results of this study are therefore reliable.

## 4. Conclusions and Implications

### 4.1. Conclusions

This study conducted a systematic literature review of research on VR promoting SEL in children and adolescents from 2013 to 2023, and a meta-analysis of 15 empirical research with sufficient data. The results of the review were used to answer the research questions raised. First, when VR is applied to SEL for children and adolescents, there is a heightened focus on mental disorders, particularly ASD. Second, the most common type of VR is simulation, and high levels of fidelity and interactivity account for the largest proportion of VR, which provides opportunities for children to practice their social and emotional skills. In addition, while one of the characteristics of VR is immersion, computers are still the most used equipment and have lower immersion. Third, learning objectives were categorized, and we found that most studies focused on social interaction skills in SEL—that is, on children’s ability to communicate and cooperate. Finally, the results of the meta-analysis showed that VR interventions had a medium effect on SEL in children and adolescents, and the factors of disease type and instructional task had a significant moderating effect on the instructional use of VR.

The capacity of VR to promote social emotional learning for children and adolescents are attributable to two essential technical affordances: a sense of presence and embodied interaction. The high-fidelity displays, realistic communication, and achievable actions endow learners with computer-mediated presence or co-presence, resulting in learning benefits such as experiential, contextual, and collaborative learning [65]. For instance, in simulated virtual scenarios (e.g., school, public places, nature), children can perceive the emotional state of virtual characters and comprehend the social cues through observation and recognition of their facial expressions, tones, and postures. This simulation helps children correctly identify others’ emotions and intentions and respond appropriately. Students in VR can also engage in social activities such as conversations and greetings with virtual avatars or real peers, simulating real-world social behavior, and thus exercising their communication and problem-solving skills.

Moreover, VR allows children repeated practice in a private and safe environment, and children interact socially in VR scenes as if they were playing a game. Studies have shown that pretending to play games improves the social and emotional competence of children with ASD [66]. The relatively lower VR efficacy for TD children may be that they have more opportunities to interact with others in the real world. Furthermore, VR interventions with more than 30 attempts tend to have higher efficacy. This is because social and emotional skills require long-term cultivation and are not acquired instantaneously. Unsurprisingly, virtual worlds tend to have better outcomes compared to simulations. This is because virtual worlds not only mimic real-life scenarios but also incorporate social elements, enabling students to engage in more open communication within the environment.

### 4.2. Practical Implications

The primary objective of this review was to describe and evaluate the utilization of VR intervention to enhance SEL in children and adolescents. The findings have practical implications for VR instructional practices. To optimally leverage the benefits of VR for improved instructional outcomes, concrete and feasible recommendations are therefore provided.

First, the present study advocates for a greater utilization of VR technology in promoting SEL in children with ASD. The findings demonstrate that VR interventions are more efficacious in children with ASD than in TD children. This is primarily because children with ASD possess a greater potential for development in social and emotional competence relative to TD children. TD children have reached an optimal level, a phenomenon that is consistent with the exposition of Amat and Zhao [20]. However, this does not signify that the SEL of TD children is of secondary importance. This position arises from a comparison of children with ASD to TD counterparts [43]. Children with mental disorders display more salient shortcomings in SEL, which necessitate multiple interventions to achieve effective amelioration. Moreover, the prevalence of ASD in children has continued to rise [67,68]. The application of VR in children with ASD may thus hold great significance.

Second, the use of advanced VR technology to provide children with improved learning support is recommended. Research has shown that the effectiveness of using computers for VR teaching is relatively limited, but the most commonly used device in current research on VR applications to promote SEL in children is still the computer. Although there are advantages to using computer equipment, such as lower cost and convenient portability, its immersive ability is limited, and it does not provide a strong sense of presence. Meanwhile, it is not surprising that projection achieved the best learning outcomes. Projection equipment was used in the cave environment, and this new system can increase interactivity [43], i.e., children do not need to wear heavy helmets; teachers can participate in the intervention process; and actions in the physical world can be displayed in real time on the projection screen, which helps children to correctly understand their social behaviors. Moreover, the virtual world type of VR teaching works best. Virtual worlds not only simulate daily life in the physical world, but also allow children exploration of the environment and engagement in open communication, thereby enabling them to safely practice their social skills [32,42].

Third, a flexible selection of pedagogy when applying VR technology in teaching is recommended. Our findings indicate that there was no significant heterogeneity among types of pedagogy, which means that the differences in instructional effectiveness between different pedagogies are not significant. This means teachers can select the most suitable pedagogy based on the instructional content and the individual characteristics of students. At the same time, teachers should actively attempt to combine multiple pedagogies to adapt to and match diverse learning activity needs. It is worth noting that there was no significant heterogeneity among various VR technologies, which provided teachers with more flexibility when making decisions about VR modalities for teaching. These two important findings jointly emphasize the significance of focusing on the integration of VR technology and pedagogy. When using VR to develop children’s social and emotional competence, teachers need to strive to combine various pedagogies with VR technology to improve teaching quality.

Finally, this paper provides recommendations for the development of VR technology that targets products designed for SEL applications among children and adolescents. Specifically, in line with the perspectives put forth by Satu et al. [69], we recommend that developers consider producing VR products that can be used for assessment rather than solely for intervention purposes. This can be achieved, for example, by creating VR scenarios that explore and examine children’s social and emotional coping abilities and reactions. In terms of product functionality, there are constraints on the data that can be captured by VR systems, which has led researchers to rely on other external tools to obtain data. This may interfere with the children’s learning process and hinder comprehensive analysis based on the learning data generated by VR environments. Developers should therefore strive to enhance the functionality of VR systems to allow the comprehensive and accurate collection and recording of data on children’s behaviors and responses. The openness of communication and interaction methods within VR should also be enhanced. Currently, most VR applications interact using preset dialog trees, which limits the ways and content that children can engage within the virtual environment. To address this issue, developers should consider implementing more flexible interaction methods, such as embedding generative artificial intelligence or allowing real-time human responses. These measures would facilitate a more natural and open communication experience for children within the virtual environment.

### 4.3. Research Implications

After integrating and analyzing the findings, some limitations in the current body of research were identified in terms of research type, research design, and data collection. Targeted suggestions for future research are therefore presented to promote more comprehensive and balanced development in this field. First, researchers should conduct diverse studies. The research results showed that experimental research accounts for the largest proportion of studies conducted. Although experimental research can test the results of VR instruction from a statistical perspective, other research methods are still necessary to enrich the field. For example, case studies can be conducted to analyze how VR affects children in greater depth, and qualitative research could be conducted to understand the views and suggestions of children and parents on the use of VR.

Second, carefully designed experimental procedures should be implemented. Most current papers employed a pre-post design, which has low internal validity. Future research should overcome challenges such as the small number of participants and adopt rigorous group experiments that would enable comparison of the effects of VR application in children with TD and children with other disorders, as well as exploration of which VR interventions are suitable for addressing specific social and emotional skill deficits [70]. This approach would provide more specific references for follow-up studies.

Third, the diversity and integrity of the research datasets should be enhanced. Researchers should not only collect quantitative data but also supplement their findings with qualitative data. When conditions permit, physiological data should be used as much as possible, because it is objective and real, which makes it very valuable for reference. In terms of data reporting, our review found that although there were many experimental studies, only a small percentage of the data met the requirements for meta-analysis. Researchers should report all key data collected, especially those required for reviews, to make possible subsequent reviews and meta-analyses.

Fourth, future research should emphasize the role of the family in the development of children’s social and emotional competence. In terms of application context, most VR scenarios designed by researchers were for schools, and the researchers did not focus on using VR to improve communication between children and their parents. While most researchers focused on the effectiveness of VR interventions, it is also important to consider factors such as parental attention to children’s emotional well-being, the quality of parents’ marital relationship, and the level of communication between home and school. Therefore, future researchers are recommended to explore the impact of family factors and their mediating and moderating effects of the family in promoting children’s SEL through VR, so that technology can better serve as a bridge between families and schools.

Finally, future researchers should conduct cost–benefit analyses. Although VR may prove to be effective in numerous areas, the purchasing of VR equipment and developing VR scenarios remain quite expensive. Most current research fails to account for the costs associated with utilizing VR. A cost–benefit analysis would clearly demonstrate the costs and benefits in terms of money and labor, thus enabling individuals to make the most suitable choices.

## Figures and Tables

**Table 1:** Inclusion and exclusion criteria.

Inclusion Criteria	Exclusion Criteria
Published from January 2013 to May 2023	Publication date of the article is not within this range
Written in English	Written in other languages
Participants are children or adolescents	Participants over 18 years of age
Empirical studies	Non-empirical studies and literature reviews
Participants use VR technology to learn	VR is not used during the intervention
Research focuses on one or more social emotional learning skills	Research does not focus on social emotional learning

**Table 2:** Code table content introduction.

Category	Code	Description
Metadata	Title	Full title
	Authors	Author names
	Year	Publication year
	Source	Journal information
	Article type	Journal/conference
	Country/region	Experimental location
Participant characteristics	Age	Age range of participants
	Gender	Male only/female only/mostly male/mostly female/Co-Ed
	Grade level	Preschool/primary school/secondary school
	IQ	Below 80/more than 80
	Disorder type	Autism spectrum disorders/neurological disorder/social anxiety disorder/ADHD/TD
Research design	Research type	Experimental/quantitative/qualitative/mixed method/survey/design-based research
	Sample size	Number of participants
	Number of interventions	Number of VR interventions
	Intervention duration	Total duration of the intervention
	Data collection	Content tests/surveys/interviews/physiological indexes/video recordings/score/observations/other
Technological features	Technological type	Simulation/game/virtual world
	Equipment	Computer/head-mounted display/glasses/projector
	Immersion level	Full immersion/semi-immersion
	Interaction level	None/low/medium/high
	Fidelity	None/low/medium/high
Instructional design	VR function	Content delivery/practice/engagement
	Pedagogy	Game-based learning/collaborative/experiential learning/direct instruction
	Learning objectives	Social interaction skills/social knowledge/positive social behavior/emotional distress/attitudes toward self and others
	Scenario	Room/school/bus/park/natural environment/game
	Task	Social task/cognitive task (low/high/metacognition)/psychomotor task
	Theoretical foundation	Record if mentioned

**Table 3:** Overall effect of virtual on social and emotional learning in children and adolescents.

Model	No. of Studies	Hedge’s *g*	95% Confidence Interval		Test of Null		Heterogeneity			
			Lower Limit	Upper Limit	Z	*p*	Q	df	*p*	*I* ^2^
Fixed	31	0.387	0.317	0.457	10.786	0.000	120.053	30	0.000	75.011
Random	31	0.378	0.226	0.531	4.861	0.000				

**Table 4:** Moderation analysis of selected experimental studies.

Moderator	*k*	*g*	95% CI	*Q_B_*	*p* Value
Number of interventions				1.525	0.677
1	11	0.252	[−0.050–0.553]		
2–10	6	0.354	[0.249–0.460] ***		
11–30	9	0.346	[−0.033–0.726]		
>30	5	0.698	[0.050–1.347] *		
Disorder type				28.117 ***	0.000
ASD	19	0.469	[0.254–0.684] ***		
SAD	1	−0.854	[−1.319–−0.388] ***		
Social problem	2	0.543	[0.134–0.951] **		
Mixed	3	0.410	[0.162–0.658] **		
TD	6	0.262	[0.010–0.515] *		
Technological type				2.905	0.234
Game	7	0.061	[−0.420–0.542]		
Simulation	20	0.370	[0.218–0.523] ***		
Virtual world	4	0.822	[0.056–1.588] *		
Equipment				2.682	0.443
Computer	17	0.275	[0.074–0.476] **		
Glasses	3	0.455	[0.212–0.698] ***		
HMD	7	0.387	[0.088–0.687] **		
Projector	4	0.822	[0.236–0.506] *		
Interaction level				1.597	0.660
Low	2	0.333	[−0.101–0.767]		
Medium	3	0.337	[0.216–0.459] ***		
High	22	0.312	[0.110–0.515] **		
None	4	0.822	[0.239–0.440] *		
Fidelity				2.824	0.588
Low	3	−0.167	[−0.979–0.645]		
Medium	3	0.410	[0.162–0.658]		
High	14	0.348	[0.141–0.555]		
None	3	0.483	[0.262–0.704]		
Not mentioned	8	0.498	[0.024–0.973]		
Theoretical basis				2.508	0.113
Yes	4	0.545	[0.372–0.719] ***		
No	27	0.346	[0.169–0.522] ***		
Task				15.126 *	0.010
Social task	12	0.622	[0.370–0.875] ***		
Cognitive task (low)	10	0.190	[−0.104–0.521]		
Cognitive task (high)	1	−2.124	[−3.759–−0.489] **		
Psychomotor task	3	0.120	[−0.885–1.126]		
Mixed	3	0.337	[0.216–0.459] ***		
Not mentioned	2	0.495	[0.205–0.785] **		
Function				1.683	0.413
Practice	26	0.322	[0.170–0.474] ***		
Engagement	1	0.438	[−0.117–0.993]		
Content deliver	4	0.391	[0.056–1.588] *		
Pedagogy				2.618	0.454
Experiential learning	19	0.360	[0.204–0.515] ***		
Game-based learning	7	0.082	[−0.427–0.591]		
Collaborative learning	1	0.438	[−0.117–0.993]		
Direct instruction	4	0.822	[0.056–1.588] *		

* *p* < 0.05; ** *p* < 0.01; *** *p* < 0.001.

## Data Availability

The data presented in this study are openly available in the Mendeley Data at https://doi.org/10.17632/2rync5xszf.1.
